# Supplementary material for: Identification of photoperiod-induced specific miRNAs in the adrenal glands of Sunite sheep (Ovis aries)
Source: Front Vet Sci. 2022 Jul 22;9:888207. doi: 10.3389/fvets.2022.888207 (PMC9354845; doi:10.3389/fvets.2022.888207)
Supplement: Supplementary file 1 [file Data_Sheet_1.ZIP › Supplementary file/miRDeep2 file.zip!/miRDeep2 file/Novel_Structure/SPLP42A2/Novel_202.pdf]

Provisional ID	:	NC_019470.2_25696
Score total	:	2.1
Score for star read(s)	:	-1.3
Score for read counts	:	0
Score for mfe	:	1.8
Score for randfold	:	1.6
Score for cons. seed	:	
Total read count	:	20
Mature read count	:	20
Loop read count	:	0
Star read count	:	0

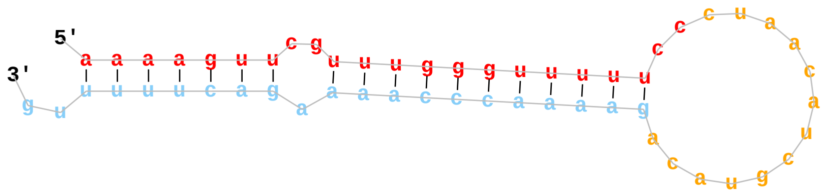

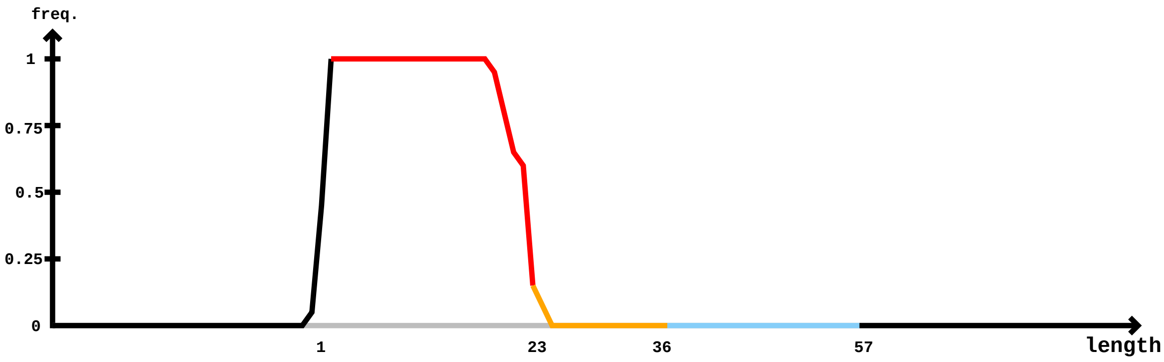

Mature **Star**

```
5'- gugaaaagucaaguauuggguuggcuaaaaaguucguuuggguuuuucccuaacaucguacagaaaacccaaaagacuuuuuggccaaccccauauauucagaaaaacaaa   -3'   exp
    ............((((.(((((((((((((((((..(((((((((((...............))))))))))).))))))))))))))))).))))...............   reads   mm   sample
    .........................Caaaaaguucguuuggguuuuuc...............................................................   1   1   seq
    ..........................aaaaaguucguuuggguuC..................................................................   2   1   seq
    ..........................aaaaaguucguuuggguuuC.................................................................   2   1   seq
    ..........................aaaaaguucguuuggguuuuuA...............................................................   3   1   seq
    ..........................aaaaGguucguuuggguuuuuc...............................................................   1   1   seq
    ...........................aaaaguucguuuggguu...................................................................   1   0   seq
    ...........................aaaaguucguuuggguuu..................................................................   1   0   seq
    ...........................aaaaguucguuuggguuuG.................................................................   1   1   seq
    ...........................aaaGguucguuuggguuuuu................................................................   1   1   seq
    ...........................aaaaguucgCuuggguuuuuc...............................................................   2   1   seq
    ...........................aaaaguucguuuggguuuuuA...............................................................   2   1   seq
    ...........................aaaaguucguuuggguuuuucG..............................................................   3   1   seq
```
